# Chromium acetate stimulates adipogenesis through regulation of gene expression and phosphorylation of adenosine monophosphate-activated protein kinase in bovine intramuscular or subcutaneous adipocytes

**DOI:** 10.5713/ajas.19.0089

**Published:** 2019-08-03

**Authors:** Jongkyoo Kim, Kiyong Chung, Bradley J. Johnson

**Affiliations:** 1 Department of Animal and Food Sciences, Texas Tech University, Lubbock, TX 79409, USA; 2 National Institute of Animal Science, Hanwoo Experiment Station, Pyeongchang 25340, Korea; 3 Korea National College of Agriculture and Fisheries, Jeonju 54874, Korea

**Keywords:** AMP-activated Protein Kinase, Bovine Adipocyte, Chromium Acetate

## Abstract

**Objective:**

We hypothesized that Cr source can alter adipogenic-related transcriptional regulations and cell signaling. Therefore, the objective of the study was to evaluate the biological effects of chromium acetate (CrAc) on bovine intramuscular (IM) and subcutaneous (SC) adipose cells.

**Methods:**

Bovine preadipocytes isolated from two different adipose tissue depots; IM and SC were used to evaluate the effect of CrAc treatment during differentiation on adipogenic gene expression. Adipocytes were incubated with various doses of CrAc: 0 (differentiation media only, control), 0.1, 1, and 10 μM. Cells were harvested and then analyzed by real-time quantitative polymerase chain reaction in order to measure the quantity of adenosine monophosphate-activated protein kinase-α (*AMPK-α*), CCAAT enhancer binding protein-β (*C/EBPβ*), G protein-coupled receptor 41 (*GPR41*), *GPR43*, peroxisome proliferator-activated receptor-γ (*PPARγ*), and stearoyl CoA desaturase (*SCD*) mRNA relative to ribosomal protein subunit 9 (*RPS9*). The ratio of phosphorylated-AMPK (pAMPK) to AMPK was determined using a western blot technique in order to determine changing concentration.

**Results:**

The high dose (10 μM) of CrAc increased *C/EBPβ*, in both IM (p = 0.02) and SC (p = 0.02). Expression of *PPARγ* was upregulated by 10 μM of CrAc in IM but not in SC. Expression of *SCD* was also increased in both IM and SC with 10 μM of CrAc treatment. Addition of CrAc did not alter gene expression of glucose transporter 4, *GPR41*, or *GPR43* in both IM and SC adipocytes. Addition of CrAc, resulted in a decreased pAMPKα to AMPKα ration (p<0.01) in IM.

**Conclusion:**

These data may indicate that Cr source may influence lipid filling in IM adipocytes via inhibitory action of AMPK phosphorylation and upregulating expression of adipogenic genes.

## INTRODUCTION

Chromium (Cr) is the 21st most abundant mineral on earth and is primarily found as divalent Cr (Cr^2+^), trivalent Cr (Cr^3+^), or hexavalent Cr (Cr^6+^) in nature [1,2]. Due to poor absorption of inorganic forms of Cr such as chromic chloride (CrCl_3_) and chromic oxide (Cr_2_O_3_) [3–5] organic sources of Cr such as Cr picolinate, Cr propionate, Cr methionine, and Cr acetate (CrAc) have been widely researched in both humans and in various livestock species. Chromium propionate is the only dietary Cr source approved by the Food and Drug Administration (FDA) for use in ruminants in the U.S [6]. It can be included in the diet at a maximum dose of 0.5 mg/kg of diet dry matter (DM) for cattle, 0.2 mg/kg of diet DM for poultry, and 0.2 mg/kg of diet DM for swine [7]. Because Cr interacts with insulin receptors, it is highly involved in lipid and protein metabolism, glucose oxidation, gluconeogenesis, and fatty acid synthesis [2,8–10].

As reported by numerous studies in the previous 60 years, Cr supplementation is related to improved carbohydrate metabolism in both humans and animals. The Cr has been shown to improve insulin signaling and subsequently induce glucose and amino acid uptake in insulin sensitive tissues such as skeletal muscle and adipose tissue [11]. Intake of Cr increased glucose uptake by enhancing insulin responsiveness through various signaling pathways [8,12]. The impact of Cr source on adenosine monophosphate-activated protein kinase (AMPK) activity has also been reported *in vitro* and *in vivo* [12]. Variation of phosphorylated-AMPKα in muscle and adipose tissue can trigger metabolic changes that switch the cell from metabolic processes geared toward energy storage to energy consuming pathways [13]. The effect of Cr and alteration of body composition is variable across animal species. In swine, many have reported that feeding Cr increases the percentage of lean tissue but decreases percentage of fat accumulation [14]. Tokach et al [15] reported that Cr propionate upregulated adipogenic gene expression and resulted in enhancing adipogenic differentiation in bovine intramuscular (IM) adipocytes. It has also been reported that Cr supplementation in lactating Holstein dairy cows increased the net synthesis of fatty acids in various adipose tissue depot [16]. Therefore, we hypothesized that Cr may differentially regulate the AMPK signaling pathway in IM and subcutaneous (SC) adipocytes resulting in increased lipid accumulation between the two different adipose tissue depots.

## MATERIALS AND METHODS

### Bovine preadipocyte isolation

Bovine IM and SC adipose tissue were collected from between the 10th and 13th rib of longissimus dorsi (LD) muscle from four 16-mo old crossbred steers (predominantly Angus, 474.5 ±50.2 kg ) following the method previously described [15]. The LD muscle was transported to the Texas Tech University Meat Science and Muscle Biology laboratory in phosphate-buffered saline (PBS: 0.76 M NaCl, 0.3 M NaH_2_PO_4_, pH 7.2) that contained 3× antibiotic-antimycotic. The IM and SC tissues were then finely minced and incubated in Dulbecco’s modified Eagle’s medium (DMEM, Gibco, Waltham, MA, USA) with 5% fetal bovine serum (FBS, Thermo Fisher Scientific, Waltham, MA, USA), collagenase (Sigma Aldrich, St. Louis, MO, USA), and 1× antibiotic-antimycotic (Gibco, USA) for 40 minutes. Digested adipose tissues were then filtered through a 250-μm filter, and the suspension centrifuged at 2,000×g. After discarding the supernatant and lipid layer, the pellet was then washed three-times using DMEM. The pellet was then re-suspended and incubated in growth media composed of DMEM, 10% FBS and 1× antibiotic-antimycotic at 37°C under a humidified atmosphere of 95% O_2_ and 5% CO_2_.

### Bovine preadipocyte cultures

Bovine IM and SC preadipocytes were cultured in DMEM containing 10% FBS until reaching ~70% to 80% confluency. Differentiation media contained 5% FBS, insulin (10 μg/mL), dexamethasone (1 μM), ciglitizone (10 μM), and oleic acid (100 μM) and was used to induce differentiation of both IM and SC cells [15]. At the same time, different doses of Cr, 0 (differentiation media only; CON), 0.1, 1.0, and 10.0 μM as chromium acetate (CrAc; Sigma Aldrich, USA) were added to the differentiation media. After 96 h of incubation, cells were harvested to analyze gene expression and protein levels.

### Morphological analysis

Oil-red-O (ORO) and hematoxylin staining were both used to determine the accumulation of lipid droplets. Cells were fixed with 10% neutral buffer formalin. After cells were rinsed with distilled water, fixed cells were then stained with 0.5% ORO solution (Sigma Aldrich, USA) in the dark for 20 min, then washed with 60% propylene glycol (Sigma Aldrich, USA). Harris’ hematoxylin (Sigma Aldrich, USA) was used to stain for nuclei in the dark for 3 min. Cells were then coated with glycerol and kept in the dark until the imaging analyses. The IM and SC preadipocytes were identified by the presence of lipid droplets contained in the cytosol, which were changed to a red color via ORO staining. All cells were viewed with a Nikon Eclipse Ti-U microscope (Nikon Instruments; NY, USA). Images were processed using NIS-Elements software (Nikon Instruments, USA). Images were analyzed using Image J program (National Institutes of Health, Bethesda, MD, USA).

### Fluorescence microscopy

Cells were grown on 3-well chamber microscopy glass slides (Cat. #: 80381, Ibidi, Fitchburg, WI, USA). Slides were fixed with 4% paraformaldehyde (Thermo Fisher Scientific, USA) for 10 min at room temperature. In order to prevent nonspecific background staining, fixed cells were incubated in 1% bovine serum albumin for 30 min. BODIPY (493/503, Thermo Fisher Scientific, USA) and BODIPY (558/568) phalloidin (Thermo Fisher Scientific, USA) diluted in dimethyl sulfoxide (Sigma-Aldrich, USA) at concentration of 1 mg/mL was used to stain neutral fat and F-actin respectively. Cells were then rinsed three times using PBS for 5 min at room temperature. In order to stain for nuclei, cells were stained with 4′,6-diamidino-2-phenylindole (Thermo Fisher Scientific, USA) for 5 min and then washed twice with PBS. The slides were imaged at a magnification of 200× using an inverted fluorescence microscope (Nikon Eclipse, Ti-E; Nikon Instruments Inc., USA) equipped with a UV light source (Nikon Intensilight Inc., USA). All images were analyzed by the NIS Elements imaging software.

### Ribonucleic acid isolation

Cultured preadipocytes were isolated using TRI reagent (Sigma-Aldrich, USA). The concentration and purity of RNA was determined with a spectrophotometer at an absorbance of 260 nm and 280 nm using a NanoDrop 1000 (NanoDrop products, Wilmington, DE, USA). An acceptable range of 1.76 to 2.05 was used for the 260:280 ratio. Ribonucleic acid (1 μg) was then reverse transcribed via TaqMan Reverse Transcription reagents and MultiScribe Reverse Transcriptase (Applied Biosystems, Foster City, CA, USA) according to manufacturer recommendations. The primers used for cDNA synthesis were random hexamers.

### Gene expression

Real-time quantitative polymerase chain reaction (7900HT Real-Time PCR System, Applied Biosystems, USA) was used to measure the quantity of adenosine monophosphate-activated protein kinase α (*AMPKα*), glucose transporter 4 (*GLUT4*), G protein-coupled receptors 41 (*GPR41*), *GPR43*, peroxisome proliferator-activated receptor γ (*PPARγ*), and stearoyl-CoA desaturase (*SCD*) relative to the quantity of ribosomal protein subunit 9 (*RPS9*) mRNA in total RNA (Table 1). Expression of *RPS9* was not different across bovine tissue samples and used as a housekeeping gene [17,18]. Therefore, *RPS9* was used as the endogenous control in order to normalize the expression of genes. Measurement of the relative quantity of the cDNA of interest was carried out using TAMRA PCR Master Mix (Applied Biosystems, USA) appropriate forward and reverse primers, and cDNA mixture. Assays were performed in triplicate determinations using and the thermal cycling parameters recommended by the manufacturer (40 cycles of 15 s at 95°C and 1 min at 60°C). Titration of mRNA primers against increasing amounts of cDNA yielded linear responses with slopes between −2.8 and −3.0. Real-time quantitative (RQ) values based on ΔΔCT^−2^ were analyzed by RQ manager (Applied Biosysytems, USA).

### Western blotting

Cultured preadipocytes were isolated using ice-cold M-PER (Thermo Fisher Scientific, USA), protease inhibitor (Sigma-Aldrich, USA), and 2 mM Na_3_VO_4_ (Thermo Fisher Scientific, USA). Homogenated cells were then mixed with an equal volume of 2×standard sodium dodecyl sulfate (SDS) sample loading buffer (Invitrogen, Waltham, MA, USA). Gradient gels were used for SDS-polyacrylamide gel electrophoresis separation of proteins. Membranes were then incubated overnight at 4°C in primary antibodies: anti-AMPKα, rabbit polyclonal (Cell signaling, Danvers, MA, USA) with dilution of 1:1,000, anti-phosphorylated AMPKα, rabbit polyclonal (Cell signaling, USA) with dilution of 1:1,000. Membranes were then incubated with a secondary antibody, Alexa-Fluor 633, goat anti-rabbit, dilution at 1:2,000 dilution for 2 h at the room temperature. After three 10 min washes, membranes were visualized using enhanced chemiluminescent substrate, Western blotting reagents (Thermo Fisher Scientific, USA), and exposure to film (MR, Kodak, Rochester, NY, USA). Density of the bands were quantified using Imager Scanner II and Image Quant TL software. To reduce the variation between blots, tissue lysates of both groups were run in a single gel. Band density was normalized according to the glyceraldehyde 3-phosphate dehydrogenase (Cell signaling, USA) content within each sample.

### Statistical analysis

Data were analyzed as completely randomized design via the Mixed procedure of SAS 9.4 (SAS Institute Inc., Cary, NC, USA). Each level of CrAc was replicated 6 times. When a significant preliminary F-test was detected, CrAc doses were separated and denoted to be different via the pairwise comparisons option of SAS. All results are reported as least-squares means. An α level of 0.05 was used to determine significance, with tendencies discussed at p-values between 0.05 and 0.10.

## RESULTS

### Morphological changes

**Figure 1:**
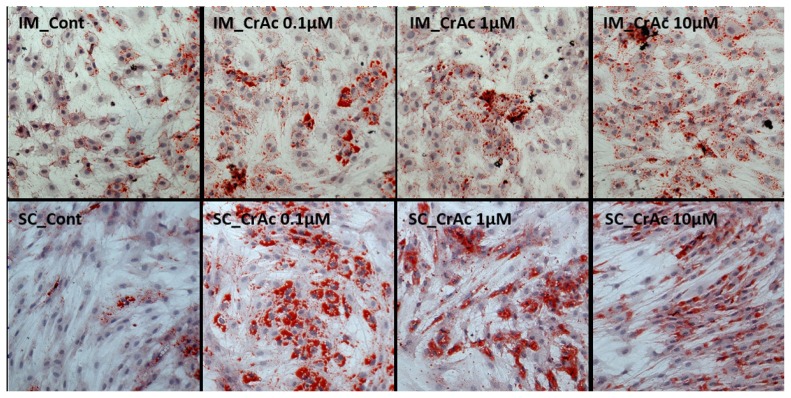
Lipid droplet visualization using oil-red-O staining. Bovine intramuscular (IM) and subcutaneous (SC) adipocytes were stained 96 h after differentiation. Cytoplasmic lipids were stained with oil-red O (red). Nuclei were stained with hematoxylin (blue). Stained cells were imaged at a magnification of 200×.

**Figure 2:**
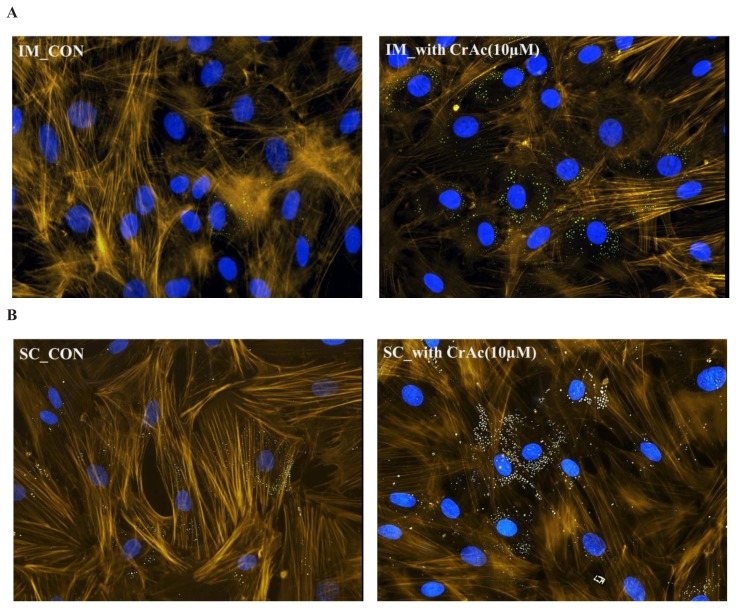
Visualization of lipid droplets in bovine intramuscular (IM) (A); and subcutaneous (SC) (B) adipose cells. Bovine IM and SC adipocytes were fixed 96 h after inducing differentiation. Lipid droplets were stained with BODIPY 493/503 (green). 4′,6-diamidino-2-phenylindole (DAPI) were used for staining nuclei (blue). F-actin was labelled with Bodipy 558/568 phalloidin (yellow). Stained cells were imaged at a magnification of 200×.

Morphological results indicated that CrAc treated adipocytes had a greater number of lipid droplets in IM and SC adipocytes compared to control (Figures 1 and 2). Bovine preadipocytes treated with CrAc contained oil-red O staining of the triacylglycerol in both IM and SC adipocytes (Figure 1). Multilocular lipid droplets developed in the cultured IM adipocytes, but unilocular lipid droplets accumulated in the SC adipocytes after 96 hours of differentiation. Lipid droplets and cell structures were visualized using BODIPY staining in IM and SC (Figure 2). Both IM and SC tended to accumulate more lipid droplets when they were incubated with CrAc (10 μM) compared to the control.

### Effects of chromium acetate on gene expression - intramuscular adipocytes

**Figure 3:**
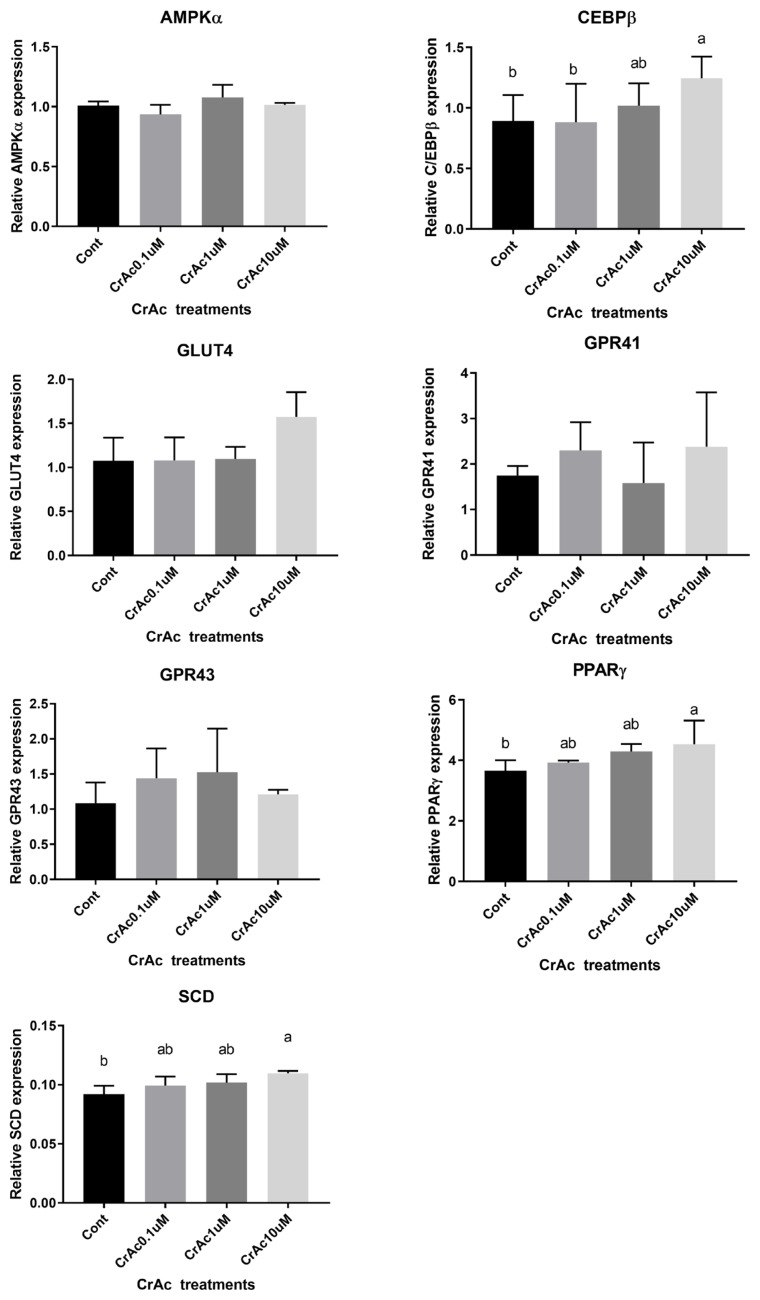
Gene expression associated with adipogenic differentiation in bovine intramuscular adipocytes (IM). Quantity of AMPKα, C/EBPβ, PPARγ, GPR41, GPR43, and SCD mRNA relative to that of ribosomal protein subunit 9 (RPS9) mRNA in total RNA isolated from cultured bovine intramuscular adipocytes were analyzed by real time-polymerase chain reaction. Addition of chromium increased (p<0.05) mRNA concentration of key transcription factors during adipogenesis, C/EBPβ, PPARγ. Expression of SCD also upregulated (p<0.01) by chromium acetate (CrAc) treatment. AMPKα, adenosine monophosphate-activated protein kinase-α; C/EBPβ, CCAAT/enhancer-binding protein β; PPARγ, peroxisome proliferated activate receptor γ; GPR41, G protein couple receptor 41; SCD, stearoyl CoA desaturase.

High concentration (10 μM) of CrAc treatment increased (p = 0.02) *C/EBPβ*, an early adipogenic transcription factor, mRNA concentration in IM adipocyte cell cultures (Figure 3). Relative mRNA expression of another key transcription factor for adipogenesis, *PPARγ*, was also upregulated (p = 0.03) when 10 μM of CrAc was included in the differentiation media compared to CON. Additionally, relative mRNA expression of the late adipogenic transcription factor, *SCD*, increased (p = 0.04) when 10 μM of CrAc was included in the differentiation media. The inclusion of CrAc in IM adipocyte cell cultures did not alter (p>0.05) mRNA abundance of *AMPKα*, *GLUT4*, *GPR41*, or *GPR43*.

### Effects of chromium acetate on gene expression - subcutaneous adipocytes

**Figure 4:**
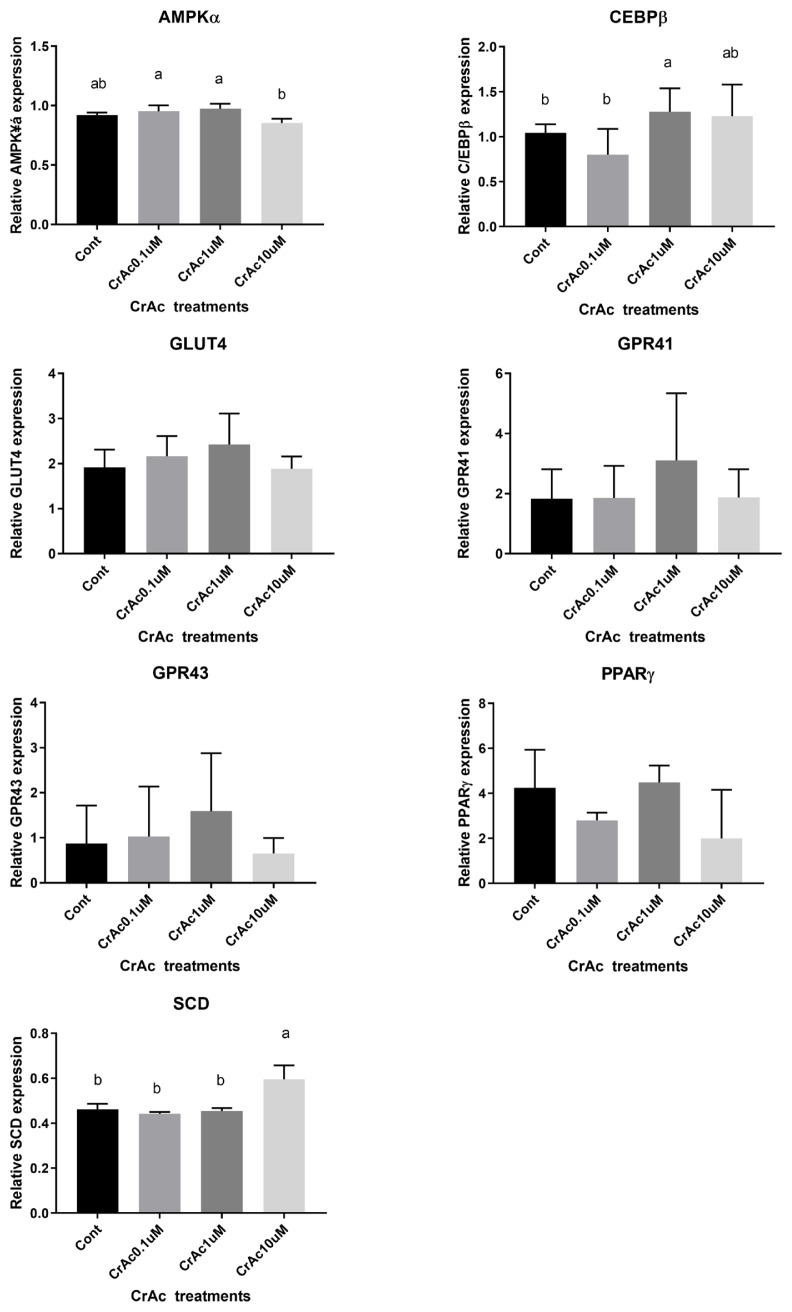
Gene expression associated with adipogenic differentiation in bovine subcutaneous adipocytes (SC). Quantity of AMPKα, C/EBPβ, PPARγ, GPR41, GPR43, and SCD mRNA relative to that of ribosomal protein subunit 9 (RPS9) mRNA in total RNA isolated from cultured bovine intramuscular adipocytes were analyzed by real time-polymerase chain reaction. Addition of chromium increased (p<0.05) mRNA concentration of the early transcription factor during adipogenesis, C/EBPβ. Expression of SCD also upregulated (p<0.01) by chromium acetate (CrAc) treatment.

In bovine SC adipocyte cell cultures, the inclusion of 10 μM CrAc in the differentiation cocktail resulted in the downregulation (p<0.02) of *AMPKα* expression compared to 0.1 μM and 1 μM of CrAc (Figure 4). However, there was no difference (p>0.05) detected between CON and 10 μM CrAc for mRNA expression of *AMPKα*. The inclusion of CrAc increased (p = 0.02) *C/EBPβ* mRNA abundance at the concentrations of 1 μM and 10 μM. Expression of *SCD* also significantly increased (p<0.01) when SC adipocyte cells were cultured with 10 μM of CrAc. Addition of CrAc did not affect gene expression of *GLUT4*. Chromium acetate also did not alter the gene expression of *GPR41* and *GPR43* in SC adipocytes.

### Phosphorylated-AMPKα and AMPKα protein level

**Figure 5:**
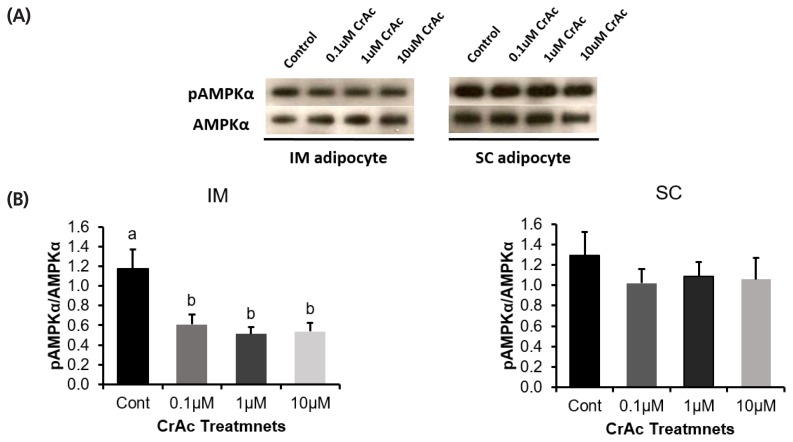
(A) Protein ration of phosphorylated AMPKα (pAMPK) to AMPKα. (B) The ratio of pAMPKα to AMPKα protein expression of bovine intramuscular and subcutaneous adipocytes are shown as determined by the western blotting technique. AMPKα, adenosine monophosphate-activated protein kinase-α.

Western blotting was used to measure the ratio of phosphorylated-AMPKα to AMPKα (Figure 5). The ratio of phosphorylated-AMPKα to AMPKα was decreased (p<0.01) 50% when bovine IM adipocytes were exposed to 0.1, 1, and 10 μM of CrAc. However, CrAc inclusion did not alter (p = 0.74) the ratio of phosphorylated-AMPKα to AMPKα in bovine SC adipocytes.

### Effects of acetate on adipose cells

**Figure 6:**
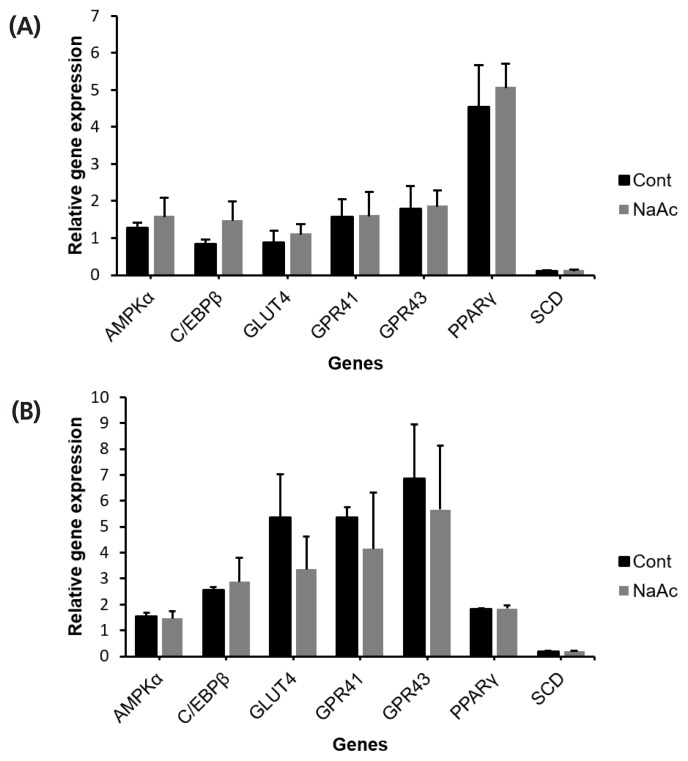
The effect of sodium acetate (NaAc) on cultured bovine intramuscular (IM) (A); and subcutaneous (SC) (B) adipocytes. Sodium acetate was added with the differentiation media to investigate the effect of acetate. After 96 h of incubation adipocytes were harvested for gene expression analysis. No significant different was found across genes (p>0.05).

To ensure the treatment effects are due to the addition of chromium source, rather than acetate, sodium acetate (NaAc) was treated on both bovine IM and SC adipocytes. Incubation with 10 μM of NaAc did not alter (p<0.05) AMPKα, C/EBPβ, SCD, PPARγ, GLUT4, and GPR43 relative mRNA abundance in IM or SC adipocyte cell cultures (Figure 6).

## DISCUSSION

Chromium plays an important role in mammals as an essential nutrient due to its substantial roles in the metabolism of various nutrients [16], insulin and glucagon regulation [9,19], and the immune system [20]. From the livestock animal standpoint, supplementation of Cr sources can influence live performance in cattle [21], and alter body composition in swine [14]. Chromium is well known to improve glucose metabolism through the enhancement of insulin sensitivity; primarily through improvement of insulin binding to its receptor, proliferation of insulin receptors, and activation of the intracellular tyrosine kinase domains contained within the insulin receptor [22]. Insulin triggers the downstream signaling pathway through phosphorylation of insulin receptor substrate 1 and phosphoinositide 3-kinases [23]. Ultimately, this results in enhancement of glucose uptake in insulin sensitive tissues by regulating the translocation of GLUT4 from the cytoplasm to the cell membrane in insulin-sensitive tissues. Tokach et al [15] reported that chromium propionate effectively increased mRNA expression of GLUT4 during differentiation of bovine IM adipocytes compared to bovine SC adipocyte. In addition, chromium (Cr^3+^) improved the GLUT4 translocation in 3T3-L1 adipocytes [24].

In the ruminant animal, it has been proposed that glucose is the primary substrate for adipogenesis in IM adipocytes, while SC adipocytes prefer to utilize the volatile fatty acid, acetate, produced via ruminal fermentation, as the primary precursor for lipid synthesis [25]. Preferential substrates differ for lipogenesis between various adipose tissue depots, and CrAc could differentially impact the lipid filling in various adipose tissue depots. Chromium markedly improves glucose uptake into insulin sensitive tissues such as adipose tissue and muscle [15]. Therefore, increasing the availability of glucose for uptake of IM adipose cells via GLUT4 can increase the accumulation of triglycerides within the IM adipocyte, but not SC adipocytes. This could explain why Cr decreases SC fat accumulation in pigs [14]. In the present study, the addition of CrAc in the differentiation media did not alter the *GLUT4* gene expression in either IM or SC adipocytes. Since only the relative mRNA expression of GLUT4 was measured, there was no way of elucidating whether or not the GLUT4 measured was contained in the cytosol or was membrane bound. The potential exists that the amount of GLUT4 that existed in cytoplasm of the adipocytes remains constant, but translocation of GLUT4 may occur when adipocytes are exposed to Cr sources.

Various studies have reported that the addition of Cr to livestock diets can have a positive effect on weight gain and variable effects on body composition [17]. A positive impact on weight seems more prominent when animals are exposed to stressful circumstances such as weaning, or co-mingling with foreign sources of cattle during marketing, and transportation [26]. In cattle, Cr has not been shown to alter body composition; however, it can decrease SC fat accumulation without altering longissimus muscle area [23]. Another study using bovine IM preadipocytes noted that addition of Cr-propionate increased expression of *PPARγ*, a key adipogenic transcription factor that may result in a compositional tissue change [15].

The induction of adipocyte differentiation involves the interplay of members of the C/EBP and PPAR families of transcription factors [27]. There are two dominant C/EBP isoforms during adipogenesis: C/EBPα and C/EBPβ. Expression of C/EBPβ in preadipocytes is initially low but greatly increases during the early stage of terminal differentiation [28]. Expression of C/EBPβ induces the activity of indispensable transcription factor, PPARγ. The PPARγ plays a critical role during adipogenic differentiation and simultaneously controls lipid metabolism-related gene expression. In lipid and glucose metabolism, synthetic PPARγ ligands including troglitazone, rosiglitazone, and pioglitazone, stimulate glucose uptake and increased insulin sensitivity in adipocytes, hepatocytes, and skeletal muscle cells [29,30]. Our data indicated that the expression of *C/EBPβ* mRNA was upregulated when SC adipocytes were exposed to 1 and 10 μM of CrAc in the differentiation media. The IM adipocytes also increased expression of *C/EBPβ* when 10 μM of CrAc was included in the differentiation media. The *PPARγ*, was highly expressed in IM when 10 μM of CrAc was added. However, the inclusion of any level of CrAc in the differentiation media of SC adipocytes did not alter *PPARγ* expression relative to CON. Therefore, Cr source may only promote the accumulation of marbling fat via stimulating key transcription factors, *C/EBPβ* and *PPARγ*. The varied responses from CrAc might be because biological and physiological differences exist between IM and SC adipose cells [31]. Recent findings reported that the IM and intermuscular adipose cells may be derived from myogenic lineage, not from adipogenic progenitors. While the overall pattern of expression of transcription factors, including C/EBPβ and PPARγ, seems to be not much different between IM and SC adipose cells [32]. The SCD is closely associated with adipogenesis and composition of muscle monounsaturated fatty acids [33]. The greatest concentration (10 μM) of CrAc increased SCD mRNA abundance in both IM and SC adipocyte cell cultures.

It is well known that AMPK plays significant role in cellu lar energy homeostasis. The activity of AMPK is regulated by various factors, such as the AMP:ATP ratio, exercise [34], and various hormones or cytokines [35]. The stimulatory role of AMPK in glucose transportation has been suggested, but there are a limited number of studies that have addressed the role of AMPK in glucose uptake in adipose cells [36]. Studies using mature adipocytes have reported that the activation of AMPK enhanced basal glucose uptake [37]. However, Salt et al [38] has shown that activation of AMPK did not affect the glucose uptake. The role of AMPK during adipogenic differentiation and lipid metabolism in adipose cells is better understood. During differentiation of preadipocytes, AMPK downregulates many key transcription factors involved in adipogenesis. Dagon et al [39] reported that the AMPK is associated with apoptosis, inhibition of lipolysis, and downregulation of the adipogenic transcription factors, PPARγ and C/EBPα in the adipogenic induced 3T3-F442a fibroblast cell line. Another study conducted using 3T3-L1 adipocytes and 5-aminoimidazole-4-carboxamide-1-β-d-ribonucleoside, which activates AMPK, suppressed the expression of PPARγ [13].

The AMPK complex is activated by phosphorylation of Thr 172 position of the α-subunit [40]. Merely measuring relative abundance of AMPK mRNA provided little insight into what is actually occurring in vivo. Thus, the ratio of phosphorylated-AMPK to AMPK must be measured. In the current study, the ratio of phosphorylated-AMPK to AMPK was decreased when CrAc was added to IM adipocytes; however, this did not occur in SC adipocytes. Conversely, chromium picolinate increased AMPK activity in L6 skeletal muscle cells [12].

Based on our findings, Cr sources likely stimulate adipo genesis both in IM and SC by stimulating key adipogenic transcription factors including C/EBPβ, PPARγ, and SCD. Our data also indicated that CrAc may deactivate the phosphorylation of AMPK and increased adipose cell development in IM but not in SC.

## Figures and Tables

**Table 1:** Primer and probe sequences for the gene expression analysis

Genes	Sequence (5′ to 3′)
*AMPKα*	
Forward	ACCATTCTTGGTTGCTGAAACTC
Reverse	CACCTTGGTGTTTGGATTTCTG
TaqMan probe	6FAM-CAGGGCGCGCCATACCCTTG-TAMRA
*CEBPβ*	
Forward	CCAGAAGAAGGTGGAGCAACTG
Reverse	TCGGGCAGCGTCTTGAAC
TaqMan probe	6FAM-CGCGAGGTCAGCACCCTGC-TAMRA
*GPR41*	
Forward	TGCTCCTCAGCACCCTGAA
Reverse	TTGGAACCCAGATGATGAGAAA
TaqMan probe	6FAM-TCCTGCGTCGACCCCCTTGTCTAC-TAMRA
*GPR43*	
Forward	GGCTTTCCCCGTGCAGTA
Reverse	ATCAGAGCAGCCATCACTCCAT
TaqMan probe	6FAM-AAGCTGTCCCGCCGGCCC-TAMRA
*PPARγ*	
Forward	ATCTGCTGCAAGCCTTGGA
Reverse	TGGAGCAGCTTGGCAAAGA
TaqMan probe	6FAM-CTGAACCACCCCGAGTCCTCCCAG-TAMRA
*RPS9*	
Forward	GAGCTGGGTTTGTCGCAAAA
Reverse	GGTCGAGGCGGGACTTCT
TaqMan probe	6FAM-ATGTGACCCCGCGGAGACCCTTC-TAMRA
*SCD*	
Forward	TGCCCACCACAAGTTTTCAG
Reverse	GCCAACCCACGTGAGAGAAG
TaqMan probe	6FAM-CCGACCCCCACAATTCCCG-TAMRA

*AMPKα*, adenosine monophosphate-activated protein kinase-α; *C/EBPβ*, CCAAT/enhancer-binding protein β; *GPR41*, G protein couple receptor 41; *PPARγ*, peroxisome proliferated activate receptor γ; *RPS9*, ribosomal protein subunit 9; *SCD*, stearoyl CoA desaturase.
